# Do replicates of independent guppy lineages evolve similarly in a predator‐free laboratory environment?

**DOI:** 10.1002/ece3.4585

**Published:** 2018-12-18

**Authors:** Kiyoko M. Gotanda, Amy Pack, Caroline LeBlond, Andrew P. Hendry

**Affiliations:** ^1^ Redpath Museum and Department of Biology McGill University Montreal Quebec Canada; ^2^ Department of Zoology University of Cambridge Cambridge UK; ^3^ Global Programs Health Standards Organization Ottawa Ontario Canada

**Keywords:** convergent evolution, experimental evolution, natural selection, parallel evolution, phenotypic trajectory, *Poecilia reticulata*, sexual selection

## Abstract

The Trinidadian guppy is emblematic of parallel and convergent evolution, with repeated demonstrations that predation regime is a driver of adaptive trait evolution. A classic and foundational experiment in this system was conducted by John Endler 40 years ago, where male guppies placed into low‐predation environments in the laboratory evolved increased color in a few generations. However, Endler's experiment did not employ the now typical design for a parallel/convergent evolution study, which would employ replicates of different ancestral lineages. We therefore implemented an experiment that seeded replicate mesocosms with small founding populations of guppies originating from high‐predation populations of two very different lineages. The different mesocosms were maintained identically, and male guppy color was quantified every four months. After one year, we tested whether male color had increased, whether replicates within a lineage had parallel phenotypic trajectories, and whether the different lineages converged on a common phenotype. Results showed that male guppy color generally increased through time, primarily due to changes in melanic color, whereas the other colors showed inconsistent and highly variable trajectories. Most of the nonparallelism in phenotypic trajectories was among mesocosms containing different lineages. In addition to this mixture of parallelism and nonparallelism, convergence was not evident in that the variance in color among the mesocosms actually increased through time. We suggest that our results reflect the potential importance of high variation in female preference and stochastic processes such as drift and founder effects, both of which could be important in nature.

## 1 INTRODUCTION

Ecological or environmental pressures that shape natural selection can be so strong that similar phenotypes will evolve in multiple independent populations exposed to similar environments, a phenomenon variously called “parallelism,” “convergence,” “predictability,” or “repeatability” (Arendt & Reznick, 2008; Clarke, 1975; Langerhans, Layman, Shokrollahi, & DeWitt, 2004; Losos, 2011; Oke, Rolshausen, LeBlond, & Hendry, 2017; Schluter, 2000; Wake, Wake, & Specht, 2011). Following the geometric perspective advocated by a number of authors (Bolnick, Barrett, Oke, Rennison, & Stuart, 2018; Stuart et al., 2017), we will use the term “parallel” when referring to evolution along similar phenotypic trajectories and “convergence” when referring to populations with initially different phenotypes that subsequently evolve more similar phenotypes. Evidence for these phenomena has been found in a wide variety of taxa ranging from viruses and bacteria (e.g., Travisano, Mongold, Bennett, & Lenski, 1995; Saxer, Doebeli, & Travisano, 2010; Wake et al., 2011) to invertebrates (e.g., Kilias, Alahiotis, & Pelecanos, 1980; Jones, Culver, & Kane, 1992), vertebrates (e.g., Losos, 1992; Langerhans et al., 2004; Romero, 2011), and plants (e.g., Wang & Qiu, 2006). However, numerous examples also exist where independent populations evolve noticeably different traits, reflecting both nonparallelism and nonconvergence, despite seemingly similar environments (Kaeuffer, Peichel, Bolnick, & Hendry, 2012; Lenski & Travisano, 1994; Oke et al., 2017; Revell, Johnson, Schulte, Kolbe, & Losos, 2007; Simões et al., 2008). Such nonparallelism and nonconvergence is likely due to unrecognized variation in important selective factors (Kaeuffer et al., 2012; Stuart et al., 2017), including sexual selection (Bonduriansky, 2011; Maan & Seehausen, 2011; Schwartz & Hendry, 2007), as well as genetic properties such as genetic drift/founder effects, variability in standing genetic variation, and genetic constraints (Elmer & Meyer, 2011; Rosenblum, Parent, & Brandt, 2014; Simões et al., 2008; Stuart et al., 2017). We were interested in exploring the extent of nonparallelism and nonconvergence in a study system where parallelism and convergence are typically emphasized.

**Figure 1:**
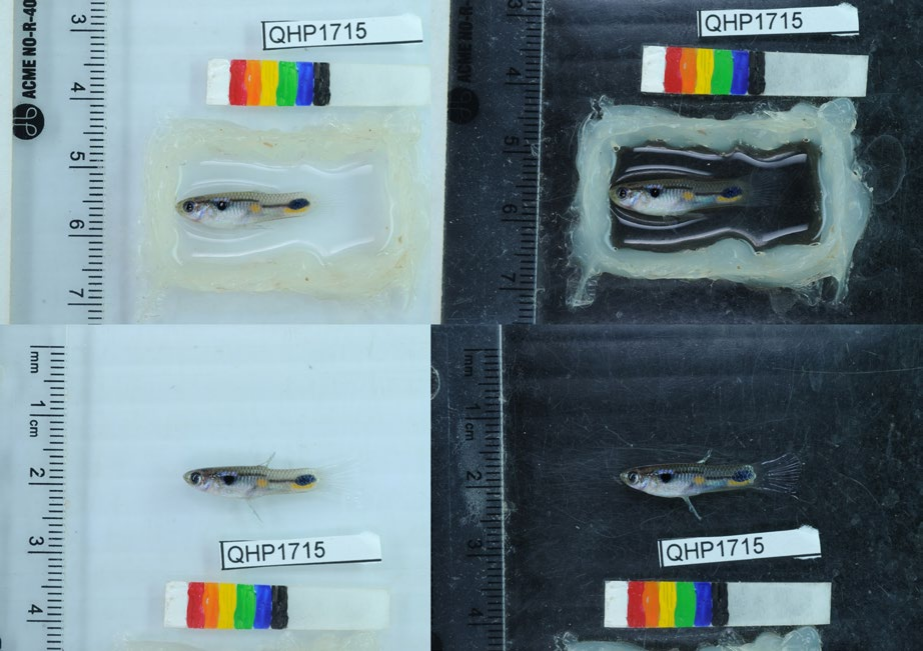
Representative photographs of a male Trinidadian guppy. The four photographs are of the same fish. Two photographs are “wet” where the individual was placed in water in a silicone well. Two photographs are “dry” where fish were gently blotted to remove excess water and their fins were spread. Each of the photographs was then photographed against a white or black background

The Trinidadian guppy (*Poecilia reticulata*; Figure 1) is considered emblematic of parallel and convergent evolution, wherein many adaptive traits have repeatedly and predictably evolved in response to strong natural selection (Endler, 1995; Magurran, 2005; Oke et al., 2017). For instance, populations experiencing different predation regimes (high‐predation vs. low‐predation) exhibit different color patterns (Endler, 1978), color amounts (Gotanda & Hendry, 2014; Winemiller, Leslie, & Roche, 1990), life histories (Reznick & Endler, 1982; Reznick, Rodd, & Cardenas, 1996), opsin genes (Sandkam, Young, & Breden, 2015), morphologies (Hendry, Kelly, Kinnison, & Reznick, 2006), parasite resistance (Dargent, Scott, Hendry, & Fussmann, 2013), and behaviors (Burns, Price, Thomson, Hughes, & Rodd, 2016; Houde & Endler, 1990; Kelley & Magurran, 2003; O'Steen, Cullum, & Bennett, 2002). However, various studies have also identified nonparallel aspects to this divergence, including for color (Kemp, Reznick, Grether, & Endler, 2009; Millar & Hendry, 2012), female preferences (Endler & Houde, 1995; Houde & Endler, 1990), life history (Fitzpatrick, Torres‐Dowdall, Reznick, Ghalambor, & Funk, 2014), morphologies (Odell, Chappell, & Dickson, 2003), parasite resistance (Pérez‐Jvostov, Hendry, Fussmann, & Scott, 2015), and behavior (Jacquin et al., 2016). Importantly, nonparallel and nonconvergent evolution in response to these predation regimes seems to involve both the direct effects of predation and a host of other associated factors such as environmental productivity, parasitism, and competition (Bassar, Lopez‐Sepulcre, Reznick, & Travis, 2013; Pérez‐Jvostov, Hendry, Fussmann, & Scott, 2016; Simon et al., 2017; Travis et al., 2014).

Beyond correlative analyses of natural populations, the guppy system is particularly amenable to testing the predictability of evolution by means of experiments (Reznick & Ghalambor, 2005). In the often‐used classic approach, guppies from high‐predation populations are introduced into low‐predation populations, after which low‐predation phenotypes typically evolve within a few generations (e.g., Endler, 1980; Reznick & Bryga, 1987; O'Steen et al., 2002; Gordon et al., 2015, 2009). In the case of male color, females generally prefer males with more color, especially carotenoid and structural colors (Endler, 1984; Gordon et al., 2015; Grether, 2000; Houde, 1997; Kodric‐Brown, 1985, 1989, 1993 ), whereas predators often kill more colorful males at higher rates (Endler, 1980; Magurran, 2005; but see Weese, Gordon, Hendry, & Kinnison, 2010). Combining these and other correlated aspects of selection, high‐predation males introduced to low‐predation sites typically evolve increased color in short order (Endler, 1980; Gordon et al., 2015). Yet similar introduction experiments have documented considerable idiosyncrasies in that high‐predation guppies introduced to low‐predation sites do not always evolve the same color phenotypes (Fitzpatrick et al., 2017; Karim, Gordon, Schwartz, & Hendry, 2007; Kemp et al., 2009), and—indeed—different natural (nonexperimental) low‐predation guppy populations often differ considerably in male color (Gotanda et al., 2013; Kemp, Reznick, & Grether, 2008; Millar, Reznick, Kinnison, & Hendry, 2006; Weese et al., 2010). These nonparallel and nonconvergent patterns could be due to the aforementioned environmental (differences in selection among “replicate” locations) or genetic (drift, founder effects, starting variation, and mutation) causes.

Isolating the environmental and genetic contributions to nonparallelism and nonconvergence requires particular experimental designs. To isolate the environmental contribution, one approach is to introduce guppies from a single population into multiple new locations. Employing this design, Gordon et al. (2015) found mostly parallelism, but with some nonparallel components, in male guppy color evolution. To isolate the genetic effect, the flip‐side approach would be to introduce multiple guppy lineages into the same (controlled and replicated) environment. Given that environments are never identical among “replicate” locations in nature, laboratory experiments are frequently used for this purpose. In the guppy system, Endler (1980) classically conducted a large‐scale laboratory experiment where he mixed guppies from 18 source populations of varying predation regimes for several generations and then introduced them into multiple mesocosms. The study documented parallelism in male guppy color evolution, with males in all mesocosms evolving increased color in six months’ time—corresponding to only a few guppy generations. However, by creating an admixed population, this experiment could not assess whether independent lineages, and thus lineage‐specific genetic backgrounds, would phenotypically converge, nor whether replicates of the same lineage would evolve in parallel.

We here extended Endler's (1980) approach by conducting a laboratory experiment that introduced each of two independent high‐predation guppy lineages (to assess convergence) into each of three laboratory mesocosms (to assess parallelism). We also introduced a mixture of the two lineages into each of three other mesocosms to generate insights into whether admixed populations might show qualitatively different patterns than “pure” populations, possibly due to an increase in genetic variation on which selection could act. After a four‐month acclimation period, we tracked the male guppy color in all nine mesocosms for an additional eight months to ask three questions about parallelism and convergence. i) Does male guppy color increase through time as had been previously observed (Endler, 1980)? ii) Do phenotypes converge when different lineages are placed into similar environments? iii) Do guppies in replicate mesocosms show parallel changes in color, particularly when they are from the same lineage?

## 2 METHODS

### 2.1 Field sampling and laboratory maintenance

In February of 2009 and 2010, male and female guppies were collected from two high‐predation (HP) populations where guppies coexist with dangerous piscivorous predators (Endler, 1980; Gotanda et al., 2013). The Aripo River is in the Caroni drainage, and our specific collection site (10 38’55”N 61 13’28”W) has been the subject of numerous studies of guppy evolution (e.g., Reznick & Endler, 1982; O'Steen et al., 2002; Schwartz & Hendry, 2007; van Oosterhout et al., 2007; Kemp et al., 2008). This particular high‐predation population is where Endler (1980) collected guppies and moved them upstream and above waterfalls to establish a low‐predation population where they rapidly evolved higher color. The Quare River is in the Oropuche drainage, and our collection site (10 39’48”N 61 11’38”W) has also been the subject of numerous studies of guppy evolution (Grether, 2000; Grether, Hudon, & Millie, 1999; Kemp et al., 2008; Millar & Hendry, 2012; Rodd, Hughes, Grether, & Baril, 2002). The guppies in these two drainages have been isolated from each other for approximately one million years and are genetically distinct (Alexander, Taylor, Wu, & Breden, 2006; Carvalho, Shaw, Magurran, & Seghers, 1991; Fraser, Künstner, Reznick, Dreyer, & Weigel, 2015; Shaw, Carvalho, Magurran, & Seghers, 1991), yet they are exposed to common piscivores, such as the pike cichlid, *Crenicichla sp*., and the two‐spotted sardine, *Astyanax bimaculatus* (Kenny, 1995; Magurran, 2005; Phillip, 1998; Phillip & Ramnarine, 2001; Seghers, 1973). These two guppy populations thus allowed us to consider the potential role of different ancestral lineages on the evolution of guppy color.

**Figure 2:**
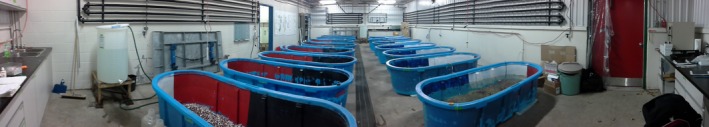
Photograph of the experimental facilities at the MacDonald Campus of McGill University

The guppies were captured from these sites with butterfly nets and immediately transported to our field station in Trinidad, where they were maintained on flake food for several days to weeks. They were then live‐transported to our facilities at the Macdonald Campus of McGill University (Figure 2). Upon arrival, guppies were separated by sex, treated with Polyguard (Seachem, Madison, GA) to eliminate parasites and other diseases, and maintained separately by source population. During this period, the fish were housed in glass aquaria (2.5 gallon), maintained on a 12:12 photoperiod, and fed ad libitum a combination of brine shrimp, liver paste, and flake food. Randomly selected males were periodically introduced for a duration of 24 hr into tanks housing only females. This movement of males was done within each population (only males and females from the same population were in a tank together) every four days to facilitate offspring production and increase the effective population size (Nakatsuru & Kramer, 1982). The F_1_ laboratory‐reared offspring of the F_0_ wild‐caught fish were maintained in a similar manner (separated by sex when mature and maintained separately in glass tanks), as were their F_2_ laboratory‐reared offspring.

### 2.2 Mesocosm setup and maintenance

Nine experimental mesocosms (each a 300‐gallon plastic cattle trough; ~1 m x ~2.5 m dimensions) were used for the experiment (Figure 2). Substrate color in Trinidadian rivers is highly variable (Endler, 1978), such that no single color would accurately reflect natural substrate variation. We therefore painted each of the four quarters of each mesocosm in a different color (white, black, red, and blue). We recognize that this is not representative of what is found in nature nor of previous experiments (e.g., Endler (1980) used different size and color of gravel). However, our goal was to understand phenotypic divergence among replicates within and among populations, and thus, it was crucial to have each mesocosm to be as similar as possible, even if it was not representative of nature nor of previous experiments. The four different colors also create different viewing environments, yet the guppies did occupy the entire mesocosm, indicating male guppies were viewed under a variety of spectral conditions (AP and KMG, personal observation). In each case, one coat of aquarium‐friendly paint (Krylon Fusion for Plastic) was applied to each mesocosm and allowed to cure for a minimum of one week. To each mesocosm, we also added four centimeters depth of mixed gravel (brick red and white), two sponge bubble filters, and six fake plants made of fabric and plastic. All products are commercially available at retail aquarium stores. Water used in the mesocosms (40 cm depth) was tap water conditioned with Start Right (Jungle Labs) or NovAqua (Kordon LLC), both of which remove ammonia, nitrates, nitrites, and chlorine. We also added Biozyme (Aquarium Products), which helped seed the water with beneficial bacteria and enzymes. One week prior to the start of the experiment, 10 male stock guppies were introduced into each mesocosm to further promote the buildup of microbiota. All males were removed 24 hr prior to the experimental introductions.

Our goal with this experimental setup was to replicate environmental conditions among mesocosms as closely as possible. Although we cannot rule out subtle differences in initial starting conditions or the subsequent accumulation of those differences, all mesocosms were treated the same and are at least much more similar to each other than would be “replicates” of “similar” environments in nature. Given our specific interest in (non)parallelism/(non)convergence, our focus was on comparing the different mesocosms to each other, rather than attempting to infer a specific causal reason for any particular evolutionary change. For this reason, “control” mesocosms were not implemented, nor would a “control” environment pertaining to this experimental design be clear.

Each mesocosm was seeded with 10 females and 10 males that were a mix of *F*_0_, *F*_1_, and *F*_2_ fish in roughly equal proportions. The number of introduced fish was chosen to balance the desire to mimic natural bottlenecks during colonization of low‐predation sites in nature with the desire to not too severely limit genetic variation. The mixture of generations was necessary because not enough fish from any single generation was available to implement the full experimental design. Introductions were done in three blocks (November 2010, March 2011, and July 2011), each block involving three mesocosms: one with fish from the Aripo only, one with fish from the Quare only, and one with an equal mix of Aripo and Quare fish (hereafter referred to as AxQ). Once introduced, the fish in the mesocosms were not disturbed except for regular sampling of all fish every four months. The experiment in its entirety was maintained for one year (minimum 3–4 overlapping guppy generations), which represents enough time in which to observe color evolution (Endler, 1980; Gordon et al., 2015; Reznick, Shaw, Rodd, & Shaw, 1997).

Throughout the experiment, water temperature was maintained between 19°C and 23°C by means of underwater heaters used in the spring, fall, and winter. While this temperature range is on the low end of what is found in natural populations in Trinidad (Strauss, 1990), this temperature range is well within guppy thermal tolerance and guppies now occur naturally across the globe in a variety of temperature ranges comparable to our range (Deacon, Ramnarine, & Magurran, 2011; Kent & Ojanguren, 2015). Full‐spectrum bulbs were maintained on a 12:12 photoperiod. Guppies in each mesocosm were fed twice daily with brine shrimp, flake food, or homemade liver paste. Each mesocosm received a standard minimum amount of food, with additional food scaled by visual estimates of guppy density—thus maintaining similarity to the extent possible in food per fish. Apparently sick fish were isolated and medicated in separate aquaria until they died or were healthy enough to be reintroduced into their mesocosm. Dead fish were promptly removed from the mesocosms.

### 2.3 Photographic procedure and data collection

At the time of introduction into a mesocosm, and every four months thereafter for one year, all adult fish (>13 mm standard length) were captured with aquarium nets and photographed. For the purposes of this study, only photographs of males were used. Individual fish were lightly anesthetized in a buffered solution of 0.01% Tricaine Methanesulfonate (Finquel MS‐222; Argent Laboratories Group) and placed left‐side‐up in a water‐and‐anesthetic‐filled silicone well on a piece of clear plexiglass. Photographs were then taken with a Nikon DSLR camera (D80) and a 60‐mm fixed‐length macro lens with an aperture of f/16 and a shutter speed of 1/8 s. Illumination was provided by two full‐spectrum lights with supplemental light from a Nikon Speedlight Commander Kit R1C1 flash, similar to some other studies of guppies (e.g., Gotanda et al., 2013). All photographs included a ruler and a color standard made from standard, commercially available acrylic paint (Liquitex Heavy Acrylic Paint). Each fish was photographed twice, once against a black background and once against a white background. After taking the two photographs in water, the fish was gently removed from the well, blotted dry to reduce reflection, and placed left‐side‐up directly on the plexiglass. The fish was then photographed twice more, once against a black background and once against a white background. These four photographs for each fish facilitated an accurate visual characterization of color spots, including structural colors, which are more prominent against a black background (Figure 1). After the photographs, the fish were placed in a recovery tank and, later in the same day, returned to their mesocosm. Survival rate during the above procedure was 99% across all sampling periods (KMG and AP, personal observation).

Details on data collection from digital photographs can be found in Gotanda and Hendry (2014) and are briefly outlined here. The digital photographs of male guppies were analyzed in random order by one person (AP) using ImageJ ( https://imagej.nih.gov/ij/). All four photographs were viewed simultaneously, and spot measurements were done on photographs of the “dry” fish against the white background. Sexual maturity was determined both by the standard length of the males and by confirming under a microscope a fully developed gonopodium (Houde, 1997). When more than 20 sexually mature males were present in a given mesocosm at a given time, they were haphazardly subsampled down to 20 males for analysis (Table 1), which is sufficient for quantifying mean color parameters for a population (Gotanda & Hendry, 2014).

**Table 1:** Number of males sampled for each mesocosm at each sampling month

Mesocosm	Population of origin	Month 0	Month 4	Month 8	Month 12
1	Quare	10	8	17	20
2	Aripo	9	5	15	3
3	AxQ	10	11	15	18
4	Aripo	10	17	16	2
5	AxQ	8	20	20	14
6	Quare	10	11	18	8
8	Quare	9	9	14	18
9	AxQ	10	13	15	10
11	Aripo	10	1	10	18

The first step in analysis was to categorize each spot into one of eight color categories: blue, black, fuzzy black, green, orange (including red), silver, yellow (including bronze), and violet (Endler, 1978, 1991 ; Gotanda & Hendry, 2014; Karim et al., 2007; Millar et al., 2006). These colors were then grouped into biologically relevant groups: “carotenoid” (red, orange, and yellow), “melanic” (black and fuzzy black), and “structural” (blue, green, silver, and violet). We henceforth use these group terms for convenience, while acknowledging they do not represent some variation in how these colors are produced. The following metrics were then calculated for each fish: *relative area* was the area of a given color on the fish's body relative to the fish's total body area (as a proportion), the *number of spots* was the total number of spots of a given color on the body of the fish, and *spot size* was the average size of all spots of a given color on the body of the fish.

### 2.4 Statistical analysis

All statistical analysis was performed in the R environment (version 3.3.1). Data were first transformed—relative area was arcsine‐square‐root‐transformed and spot number was square‐root‐transformed. The response variables were standardized across the entire dataset by subtracting (centering) the mean from each value and then (except for the continuous explanatory variable body size) dividing by the standard deviation. Our analyses exclude the starting Month 0 so as to reduce effects of initial plasticity that might follow introduction to the new environment. This approach also emulated Endler's (1980) evaluation of color over 6 months after a 22‐week “founding” time period. This initial plasticity could affect the phenotypic trajectories so we also repeat appropriate analyses including Month 0. To evaluate the effects of population of origin (Aripo, Quare, AxQ) and month (the three sampling times per mesocosm—Month 4, Month 8, and Month 12), using the lmer() function from the lme4 package (Bates, Maechler, Bolker, & Walker, 2015), we first ran a mixed‐model ANCOVA where the response variable was the sum of all measurements of all color groups for a given color metric (relative area or spot number), population of origin and month (ordered) were fixed factors, and mesocosm was a random effect (while accounting for the variance in the effects of month on color across mesocosms). We also ran a mixed‐model ANCOVA where the response variable was the average spot size for all color groups. We could not run a full MANCOVA using the three color groups in a single analysis for a given color metric because the model would be overparameterized.

We then ran separate, individual ANCOVAs on each color metric (relative area, spot number, average spot size) in each color group (carotenoid, melanic, structural). Population of origin and month (ordered) were fixed factors, body size was the covariate, and mesocosm was a random effect. For these analyses, we again used the lmer() function from the lme4 package (Bates et al., 2015), Anova() from the car package (Fox & Weisberg, 2011), and rand() from the lmerTest package (Kuznetsova, Brockhoff, & Christensen, 2017). To obtain *R* ^2^ values, we used the r.squaredGLMM function in the MuMIn package (Barton, 2016).

One specific prediction for convergent evolution would be decreasing variance in mean trait values, such as the amount of color, among mesocosms. That is, phenotypes might start from diverse points shaped by the different selection pressures experienced in nature (as well as stochastic founding effects in the experiment) and then converge toward a similar endpoint shaped by the similar environments they now experience in the laboratory. To test this prediction, we first determined how different the starting points for each color group and metric were by running a mixed‐model ANCOVA with mesocosm as a random factor, population as a fixed factor, and body area as a covariate. We then conducted a one‐way paired *t* test of variances among mesocosms in mean trait values between Month 4 and Month 12 to see whether variance significantly decreased. One mesocosm was removed from the analysis due to not having any variance because it had one male at Month 4 (Table 1). These analyses were performed separately for each color group (all, carotenoid, melanic, structural) for each color metric.

We next analyzed trajectories of color change through time by conducting “phenotypic trajectory analysis” (PTA) to compare the size, direction, and shape of change in phenotypic trait space (Adams & Collyer, 2009). See Adams and Collyer (2009) for detailed explanations of these measures: size (e.g., the total length of phenotypic trait change), direction (e.g., the extent to which changes in trait space are in the same direction), and shape (e.g., the extent to which stepwise trajectories share the same overall shape). We ran a PTA for each color metric (relative area, spot number, and average spot size) separately because the different units for the measurements do not allow for comparison within the same analysis (Huttegger & Mitteroecker, 2011). We ran the PTA comparing all mesocosms. We then assessed whether replicate mesocosms within a lineage had different trajectories (i.e., parallel or not) by computing pairwise comparisons within a lineage. In these PTAs, we used the three sampling points after initial introduction (Months 4, 8, and 12) and body size was included as a covariate.

## 3 RESULTS

### 3.1 Does male guppy color increase in the laboratory?

**Figure 3:**
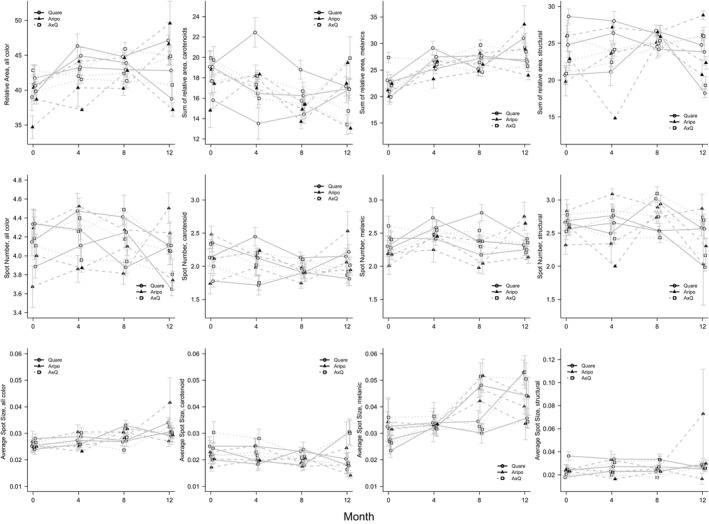
Plots showing sum of relative area (top row), spot number (second row), and average spot size (third row) through time for each mesocosm. Each plot shows color group: all color, carotenoid, melanic, and structural. Error bars denote standard error. Symbols represent the different populations of origin

If we had to highlight only one main result in our experiment, it might be that male guppy color generally increased through the course of eight months in the mesocosms (Figure 3), such that time had a strong effect on two total color metrics (spot number: χ^2^ = 15.079, *R* ^2^ = 0.203, *p* < 0.001; average spot size: χ^2 ^= 13.099, *R* ^2^ = 0.068, *p* = 0.001), although not the third color metric (relative area: χ^2^ = 0.202, *R* ^2^ = 0.275, *p* = 0.904). Results are comparable when including Month 0 and thus considering 12 months in the mesocosms, where time had a strong effect on all color metrics (relative area: χ^2^ = 17.203, *R* ^2^ = 0.279, *p* < 0.001; spot number: χ^2^ = 16.876, *R* ^2^ = 0.185, *p* < 0.001; average spot size: χ^2^ = 30.511, *R* ^2^ = 0.093, *p* < 0.001). When considering color groups and metrics individually, time had a significant effect on carotenoid relative area, melanic spot number and average spot size, and structural spot number (Table 2). Inclusion of Month 0 shows comparable results with the only difference being time also having a significant effect on melanic relative area (Table 3). Melanic color metrics all increased through time, especially average spot size (Figure 3). By contrast, carotenoid and structural color metrics showed inconsistent directions of change through time (Figure 3). Population of origin did not have a significant effect on any color metric (Table 2), mainly because differences among replicates within a given population were large in relation to the differences among populations that averaged across replicates. If we included Month 0, population of origin had a significant effect on one metric only, melanic spot number (Table 3).

**Table 2:** Results from linear mixed models for individual color metrics and groups with the three months (ordered; Month 4, Month 8, Month 12) and population as fixed factors, mesocosm as a random factor (while accounting for the variance in the effects of month on color across mesocosms), and body size as a covariate. Bold indicates significant *p*‐values

	Carotenoid		Melanic		Structural	
	χ^2^	*p*	χ^2^	*p*	χ^2^	*p*
Relative area						
Month	6.395	**0.041**	3.688	0.158	5.843	0.054
Population of origin	3.985	0.136	3.042	0.218	2.698	0.259
Body size	4.495	**0.034**	4.651	**0.031**	17.654	**<0.001**
Mesocosm	19.5	**0.002**	21.9	**<0.001**	43.5	**<0.001**
Spot number						
Month	1.645	0.439	18.164	**<0.001**	15.049	**0.002**
Population of origin	0.699	0.705	5.349	0.069	0.190	0.909
Body size	0.160	0.689	15.291	**<0.001**	1.347	0.245
Mesocosm	4.45	0.5	4.5	0.5	22.2	**0.008**
Spot size						
Month	1.509	0.470	16.855	**<0.001**	0.789	0.674
Population of origin	2.475	0.290	0.390	0.822	1.088	0.580
Body size	7.842	**0.005**	2.371	0.124	5.774	**0.016**
Mesocosm	16.0	**0.007**	8.17	0.1	20.2	**0.001**

**Table 3:** Results from linear mixed models for individual color metrics and groups with the four months (ordered; Month 0, Month 4, Month 8, Month 12) and population as fixed factors, mesocosm as a random factor (while accounting for the variance in the effects of month on color across mesocosms), and body size as a covariate. Bold indicates significant *p*‐values

	Carotenoid		Melanic		Structural	
	χ^2^	*p*	χ^2^	*p*	χ^2^	*p*
Relative area						
Month	18.091	**<0.001**	35.415	**<0.001**	6.721	0.081
Population of origin	4.861	0.088	4.974	0.83	1.867	0.393
Body size	4.882	**0.027**	6.115	**0.013**	14.576	**<0.001**
Mesocosm	20.718	**0.014**	28.732	**<0.001**	61.187	**<0.001**
Spot number						
Month	5.696	0.127	17.455	**<0.001**	15.049	**0.002**
Population of origin	0.825	0.662	20.394	**<0.001**	0.190	0.910
Body size	0.698	0.403	21.883	**<0.001**	1.347	0.246
Mesocosm	7.553	0.580	2.790	0.972	22.241	**0.008**
Spot size						
Month	2.027	0.567	35.384	**<0.001**	2.050	0.562
Population of origin	4.09	0.129	1.301	0.522	1.270	0.530
Body size	10.245	**0.001**	3.014	0.083	4.896	**0.027**
Mesocosm	18.377	**0.031**	10.925	0.281	36.92	**0.001**

### 3.2 Do phenotypes converge when different populations are placed in a similar laboratory environment?

**Figure 4:**
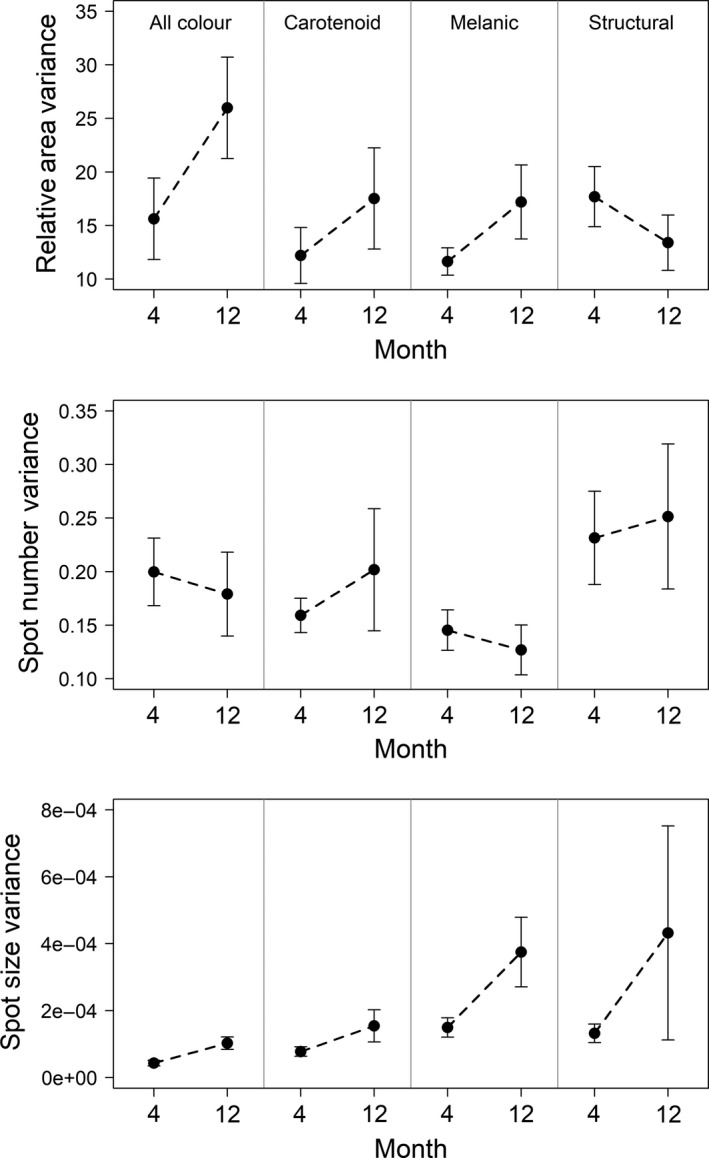
How variance among mesocosms changed from Month 4 to Month 12. Means and standard errors for variance across all mesocosms are shown. Each row is a color metric, and each column is a color group

In Month 4, at the end of the “acclimation” period, guppies in the different mesocosm differed in carotenoid relative area and spot number and also in structural spot number and average spot size (Table 4). Thus, the “starting points” for the remainder of the experiment differed among mesocosms for some aspects of male guppy color. However, the amount of variance among mesocosms did not then decrease from Month 4 to Month 12 (Table 5; Figure 4). Instead, variance appeared to increase among mesocosms for all color metrics except structural relative area and melanic spot number (Table 5; Figure 4). Thus, a decrease in variance was not statistically detected and convergence was mostly absent.

**Table 4:** Results from ANCOVA with population of origin as a fixed factor, mesocosm as a random factor, and body size as a covariate for color metrics at the start of when we formally quantified color (Month 4). Bold values indicate significant p‐values

	Total		Carotenoid		Melanic		Structural	
	*F*	*p*	*F*	*p*	*F*	*p*	*F*	*p*
Relative area								
Population of Origin	2.188	0.234	0.071	0.933	1.544	0.315	0.359	0.721
Body Size	2.655	0.107	0.088	0.767	0.837	0.363	1.497	0.224
Mesocosm	χ^2 ^= 0.574	0.140	χ^2^ = 16	**<0.001**	χ^2^ = 0.709	0.4	χ^2^ = 3.05	0.08
Spot number								
Population of Origin	0.047	0.955	0.171	0.847	0.990	0.376	0.026	0.974
Body Size	1.060	0.306	0.373	0.543	1.545	0.217	1.110	0.295
Mesocosm	χ^2^ = 6.61	**0.01**	χ^2^ = 9.26	**0.002**	χ^2^ = <0.001	1.000	χ^2^ = 6.44	**0.01**
Average spot size								
Population of origin	0.075	0.929	0.715	0.532	0.048	0.953	0.314	0.743
Body size	0.018	0.893	0.035	0.853	0.051	0.823	0.229	0.634
Mesocosm	χ^2^ = 0.535	0.5	χ^2^ = 0.711	0.4	χ^2^ = <0.001	1.000	χ^2^ = 3.81	**0.05**

**Table 5:** One‐way paired *t* test to assess if the variance at Month 4 versus Month 12 had significantly decreased. One mesocosm was removed from the analysis due to not having any variance at Month 4. The test was performed separately for each color group and color metric

		Total color	Carotenoid	Melanic	Structural
Relative area	*t*	−1.617	−1.104	−1.906	1.882
	*p*‐value	0.925	0.847	0.951	0.051
Spot number	*t*	0.547	−0.781	0.688	−0.191
	*p*‐value	0.301	0.770	0.257	0.573
Average spot size	*t*	−2.381	−1.876	−1.791	−0.895
	*p*‐value	0.976	0.949	0.942	0.800

### 3.3 Do replicate mesocosms evolve in parallel?

**Figure 5:**
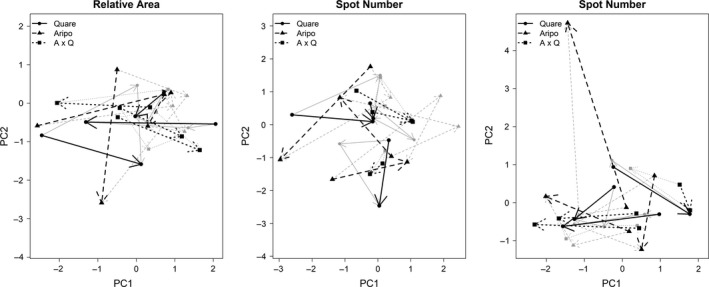
Visualization of the phenotypic trait analysis. Trajectories of PC1 and PC2 generated for all color groups in each color metric. While the phenotypic trait analysis is three‐dimensional, this image provides a two‐dimensional visualization of the phenotypic trajectories through time. Transparent arrows and symbols represent trajectories every four months (Months 4, 8, and 12); solid lines represent trajectories from Month 4 to Month 12 only

Phenotypic trajectory analysis (PTA) showed significant variation among mesocosms in the orientation (but not the size and shape) of the trait trajectories for all three color metrics (relative area, spot number, and spot size; Table 6). This outcome can be interpreted as indicating that the *magnitude* of color change through time was similar among mesocosms, but the *direction* of these changes in multivariate trait space differed among mesocosms, indicating nonparallel trait trajectory. Pairwise comparisons of individual mesocosms within a lineage showed some differences in size, orientation, and shape of phenotypic trajectories, though many comparisons were not significantly different (Tables 7, 8, 9). To aid interpretation, we visualized a 2D trajectory using the first two principal components of a PCA generated for each set of color metrics for Month 4 to Month 12 (Figure 5).

**Table 6:** Results from a phenotypic trajectory analysis (Adams & Collyer, 2009) on three different color metrics from Month 4 to Month 12 comparing individual mesocosms. All three color groups were included in the analysis. Size represents the total length of phenotypic trait change, orient is the extent to which changes in trait space are in the same direction, and shape is the extent to which stepwise trajectories share the same overall shape. Significance values were generated by residual permutations (*n* = 1,000). Bold *p*‐values indicate the phenotypic trajectory characteristic differed significantly

	Var_size_	*p*_size_	Var_orient_	*p*_orient_	Var_shape_	*p*_shape_
Relative area	0.592	0.116	1733.004	**0.001**	0.040	0.460
Spot number	0.443	0.147	1801.688	**0.001**	0.034	0.653
Average spot size	1.309	**0.032**	1691.948	**0.001**	0.60	**0.033**

**Table 7:** Results from pairwise comparisons of mesocosms within the Quare lineage of a phenotypic trajectory analysis (Adams & Collyer, 2009) on three different color metrics from Month 4 to Month 12 comparing individual mesocosms. All three color groups were included in the analysis. Significance values were generated by residual permutations (*n* = 1,000). Bold *p*‐values indicate the phenotypic trajectory characteristic differed significantly

Mesocosms	Var_size_	*p*_size_	Var_orient_	*p*_orient_	Var_shape_	*p*_shape_
Relative area						
A x B	2.471	**0.001**	131.656	0.126	0.481	0.489
A x C	1.080	0.107	142.971	0.082	0.714	0.116
B x C	1.392	**0.044**	128.832	0.691	0.738	0.083
Spot number						
A x B	0.809	0.189	64.604	0.565	0.229	0.833
A x C	0.251	0.672	103.877	0.368	0.595	0.220
B x C	0.558	0.375	47.819	0.721	0.534	0.337
Average spot size						
A x B	0.332	0.584	56.283	0.546	0.809	**0.024**
A x C	0.300	0.572	109.244	0.119	0.139	0.931
B x C	0.032	0.962	53.099	0.571	0.815	**0.023**

**Table 8:** Results from pairwise comparisons of mesocosms within the Aripo lineage of a phenotypic trajectory analysis (Adams & Collyer, 2009) on three different color metrics from Month 4 to Month 12 comparing individual mesocosms. All three color groups were included in the analysis. Significance values were generated by residual permutations (*n* = 1,000). Bold *p*‐values indicate the phenotypic trajectory characteristic differed significantly

	Var_size_	*p*_size_	Var_orient_	*p*_orient_	Var_shape_	*p*_shape_
Relative area						
A x B	1.824	**0.046**	110.157	0.306	0.198	0.875
A x C	0.360	0.733	115.454	0.234	0.684	0.256
B x C	1.464	0.196	93.600	0.407	0.589	0.511
Spot number						
A x B	0.344	0.661	82.558	0.584	0.347	0.640
A x C	0.420	0.644	105.660	0.375	0.698	0.212
B x C	0.764	0.465	166.833	**0.020**	0.859	0.052
Average spot size						
A x B	2.566	**0.018**	162.259	**0.015**	0.572	0.296
A x C	0.083	0.944	124.613	0.151	0.013	1.000
B x C	2.648	0.053	37.683	0.827	0.577	0.462

**Table 9:** Results from pairwise comparisons of mesocosms within the cross (A x Q) lineage of a phenotypic trajectory analysis (Adams & Collyer, 2009) on three different color metrics from Month 4 to Month 12 comparing individual mesocosms. All three color groups were included in the analysis. Significance values were generated by residual permutations (*n* = 1,000). Bold *p*‐values indicate the phenotypic trajectory characteristic differed significantly

	Var_size_	*p*_size_	Var_orient_	*p*_orient_	Var_shape_	*p*_shape_
Relative area						
A x B	0.072	0.899	17.414	0.974	0.443	0.495
A x C	0.738	0.265	144.451	0.073	0.440	0.528
B x C	0.810	0.123	127.077	0.166	0.725	0.084
Spot number						
A x B	0.722	0.196	38.508	0.666	0.737	0.057
A x C	0.584	0.337	53.111	0.767	0.572	0.274
B x C	0.138	0.814	86.075	0.565	0.245	0.797
Average spot size						
A x B	0.400	0.429	14.609	0.939	0.736	0.066
A x C	0.071	0.904	58.695	0.460	0.460	0.440
B x C	0.330	0.558	72.263	0.341	0.800	**0.034**

## 4 DISCUSSION

A large‐scale, yearlong experiment in which replicate laboratory mesocosms were seeded with small founding populations of guppies was used to assess how a common environment shapes (non)parallel and (non)convergent phenotypic changes. A first expectation, following from Endler (1980), was that male guppy color would increase. We did generally document such an increase, but not in the initially expected manner. Specifically, the color increase in our experiment was driven primarily by melanic color (Figure 3), whereas previous work (Endler, 1980; Gordon et al., 2015; Houde, 1997) has emphasized an increase in carotenoid and structural color. A second expectation was that phenotypes of different lineages would converge from different starting states toward similar trait optima in the common laboratory setting. Although some convergence did occur for some color variables, the overall pattern was for among‐mesocosm variance in mean trait values to *increase* through time, which suggests that divergence was stronger than convergence (Figure 4). A third expectation was that the replicate mesocosms would evolve in parallel owing to the similar laboratory environment in each mesocosm, particularly for replicates from the same lineage that share a similar genetic background. Instead, we found that color trajectories among mesocosms within a lineage were not necessarily consistent or repeatable (Tables 7, 8, 9), suggesting nonparallelism in “replicate” populations (Table 6). Given the small founding populations, we recognize that our results might simply be due to stochastic events such as genetic drift and founder effects, or it could be the result of other selective processes such as variation in sexual selection which we discuss below.

### 4.1 Increasing color, yes—but in unexpected ways

Based on previous work, we expected an overall increase in carotenoid and structural colors. These aspects of color are generally favored by females (Endler, 1984; Gordon et al., 2015; Grether, 2000; Houde, 1997; Kodric‐Brown, 1985, 1989, 1993 ) because they are thought to reflect higher fitness through (as examples) increased foraging ability (Karino & Haijima, 2004; Kodric‐Brown, 1989), better nutritional condition (McGraw, Mackillop, Dale, & Hauber, 2002), and increased parasite resistance (Houde & Torio, 1992; Kolluru et al., 2006). Thus, when guppies are brought into the laboratory, selection against investment in, and expression of, these colors is thought to be relaxed, most obviously because predators are absent (Endler, 1980). In our experiment, however, most of the phenotypic trait change was driven by an increase in melanic, rather than carotenoid or structural colors (Tables 2 and 3; Figure 3). A general explanation for this difference could be that environmental factors in our mesocosms maintain some selection against carotenoid and structural colors while simultaneously elevating selection for melanic color. The first part of this explanation seems unlikely given that, in the mesocosms, predators were absent and resources were not limiting. The second part is also uncertain. Although melanins have been linked to a variety of physiological and behavioral traits (Ducrest, Keller, & Roulin, 2008; Roulin & Ducrest, 2011), including adaptive traits such as immunocompetence (Griffith, Parker, & Olson, 2006; McGraw et al., 2002) and thermoregulation (Angilletta et al., 2006; Clusella Trullas, Wyk, & Spotila, 2007; Price, Weadick, Shim, Rodd, & Al, 2008), we have no clear evidence that any of these specific factors were more important in our mesocosms than they had been in nature.

We instead suggest that the increase in melanic color in the experiment reflected sexual selection—especially female preference—favoring these colors over carotenoid and structural colors. Early work on guppies found no correlation between female preference and melanic color (Kodric‐Brown, 1985, 1993), and subsequent work has focused on carotenoid and structural colors (reviewed in Houde, 1997; Magurran, 2005). However, some evidence does exist that females exhibit preferences for melanic color (Brooks & Endler, 2001; Endler & Houde, 1995). Moreover, recent work has found links between melanin and increased reproductive success (Gordon et al., 2015), which is consistent with increases in melanic colors in some experimental introductions (Kemp et al., 2009). Melanin also increases contrast on some backgrounds (Dale, 2006), and given the artificial color backgrounds in our mesocosms, selection for increased contrast, as opposed to a specific color group, could be occurring. At present, however, these ideas are speculative as we were unable to measure female preferences or sexual selection specifically in our mesocosms.

### 4.2 Why so little convergence?

Given the different starting points for male color in the different mesocosms, we might have expected phenotypes to converge through time due to the similar environments replicated across mesocosms. We did not find much evidence for such convergence in that variance among the mesocosm increased through time for most color metrics. This nonconvergence could reflect the typically high variation in female preferences within and among populations (Brooks & Endler, 2001; Endler & Houde, 1995; Houde & Endler, 1990; Schwartz & Hendry, 2007). That is, among‐mesocosm variation in female preference could interact with the different starting points for male color to prevent strong convergence. Certainly, color variance among the initial male guppies introduced into each of the mesocosms was high (Figure 3), indicating different color distributions on which females would be selecting. In short, our small starting populations likely resulted in “founder effects” in terms of initial male color and female preference, potentially resulting in the unexpected diversity of subsequent trajectories in male color. Additionally, some of the mesocosms went through bottlenecks where only a few males were present at certain times (Table 1), perhaps exacerbating initial bottlenecks—although this effect is likely mitigated by female storage of male sperm that means males can sire offspring long after they are dead (López‐Sepulcre, Gordon, Paterson, Bentzen, & Reznick, 2013). Importantly, such founder effects and bottlenecks are thought to accompany the natural process of colonization of upstream populations by relatively few individuals (Crispo, Bentzen, Reznick, Kinnison, & Hendry, 2006; Labonne & Hendry, 2010). Indeed, the high variance in male color and female preferences among natural upstream guppy populations (Endler & Houde, 1995; Houde & Endler, 1990; Schwartz & Hendry, 2007) could be due at least in part to such stochastic processes.

Another interpretation of increasing among‐population variance through time could be that the founding males, or their sperm (López‐Sepulcre et al., 2013), will still be present in the mesocosms by the end of the experiment. Thus, evolution toward a new optimum, combined with repeated influxes of the ancestral male DNA, could increase trait variance time—a temporal gene flow analogy to the effect of spatial gene flow increasing variance among divergent populations (Yeaman & Guillaume, 2009). However, given the degree to which among‐mesocosm variance changes through time, we think this alternative explanation is unlikely.

### 4.3 Why so little parallelism?

Despite an overall increase in color (i.e., strong parallelism at that very coarse level), specific trait trajectories differed (i.e., were not very parallel) among the mesocosms. For instance, trajectory analysis found that the orientation of change in color space was not very repeatable among mesocosms (Table 6; Figure 5). The key reason for this nonparallelism was that nearly all aspects of nonmelanic color changed in idiosyncratic ways (Figure 3). Most of this nonparallelism was among mesocosms from different lineages (Tables 7, 8, 9), as would be expected from their likely more different genetic backgrounds than replicates within a lineage. This nonparallelism we observed could, as with the nonconvergent observations described above, reflect variation among lineages and among replicates samples within a lineage in female preference for various color groups and metrics. Also, again of likely importance could be the above‐noted potential for founder effects and genetic drift due to the small founding populations and bottlenecks in the replicate mesocosms.

## 5 CONCLUSIONS

In the guppy system, deterministic natural selection is often emphasized as the primary determinant of evolutionary trajectories (Endler, 1980; Gordon et al., 2015, 2009 ; Gotanda & Hendry, 2014; O'Steen et al., 2002; Reznick & Bryga, 1987), yet, as our experiment suggests, factors such as the stochastic effects of small starting populations, bottlenecks, and drift also could play an important role—at least for male guppy color. That is, we found that the direction and magnitude of multivariate color change was highly variable among lineages, as well as among replicates within a lineage. Although some consistent selection in color (specifically melanic color) did emerge in the experiment, the strength and direction of change in other aspects of color was highly variable, likely due to variability in male color and female preference at the outset of the experiment. Hence, although guppy color evolved, it did not do so in particularly repeatable, convergent, or parallel manner. Importantly, these nonparallel and nonconvergent findings are, in fact, consistent with more recent work on guppies emphasizing nonparallelism in adaptive traits, including color (Fitzpatrick et al., 2017; Karim et al., 2007; Kemp et al., 2009; Millar & Hendry, 2012), life history (Fitzpatrick et al., 2014), morphology (Odell et al., 2003), parasite resistance (Pérez‐Jvostov et al., 2015), and behavior (Jacquin et al., 2016). In short, although natural selection is clearly a very strong force in guppy evolution, it does not always generate similar evolutionary outcomes, likely owing to variation among locations in predator diversity and abundance (Millar et al., 2006; Phillip, 1998; Torres Dowdall et al., 2012), variable sexual selection (Brooks, 2002; Endler & Houde, 1995; Lindholm et al., 2014; Schwartz & Hendry, 2007), variable genetic backgrounds (Alexander et al., 2006; Carvalho et al., 1991; Fraser et al., 2015; Shaw et al., 1991), and various stochastic effects (as we have here emphasized).

Nonparallelism in the guppy system—even in a common environment—is also consistent with a nuanced interpretation of theoretical work, and also with empirical work in other natural systems that has shown how the underlying genetic architecture of a population can greatly affect the amount and direction of phenotypic evolution (Foster & Baker, 2004; Rosenblum et al., 2014) through genetic drift/founder effects (Elmer & Meyer, 2011; Simões et al., 2008), standing genetic variation (Barrett & Schluter, 2008), genetic constraints (Weinreich, Watson, & Chao, 2005), and ancestral lineages (Alexander et al., 2006; Baxter et al., 2010; Blount, Borland, & Lenski, 2008; Lindholm et al., 2014; Price, Lovette, Bermingham, Lisle, & Richman, 2000). Parallel evolution resulting from a strong selective driver has been of theoretical and empirical interest because it provides evidence for some strong, deterministic drivers of phenotypic variation (Endler, 1986; Schluter, 2000). However, recent results from a variety of systems and theoretical models are showing that the selective and nonselective processes surrounding and shaping phenotypic evolution are complex. In short, evolutionary biologists should focus increased attention on nonparallel and nonconvergent aspects of evolution and their causes (Kaeuffer et al., 2012; Oke et al., 2017; Rosenblum, Parent, Diepeveen, Noss, & Bi, 2017; Stuart et al., 2017).

## 6 DATA AVAILABILITY

Data available from the Dryad Digital Repository: https://doi.org/10.5061/dryad.8g59456.

## CONFLICT OF INTEREST

None.

## AUTHOR CONTRIBUTION

KMG and APH conceived and designed the experiment; KMG, AP, and CL ran and maintained the experiment and helped collect photographic data; AP analyzed the photographs; KMG, AP, and APH analyzed the data; and all authors contributed to writing.
